# (3-Methyl­benzo­nitrile-1κ*N*)-*cis*-tetra­kis(μ-*N*-phenyl­acetamidato)-1:2κ^4^ *N*:*O*;1:2κ^4^ *O*:*N*-dirhodium(II)(*Rh*—*Rh*)

**DOI:** 10.1107/S1600536814016031

**Published:** 2014-07-23

**Authors:** Cassandra T. Eagle, Fredricka Quarshie, Kevin M. Cook

**Affiliations:** a Department of Chemistry, East Tennessee State University, PO Box 70695, Johnson City, TN 37614, USA

**Keywords:** crystal structure

## Abstract

The complex molecule of the title compound, [Rh_2_{N(C_6_H_5_)COCH_3_}_4_(NCC_7_H_7_)], has crystallographically-imposed mirror symmetry. The four acetamide ligands bridging the dirhodium core are arranged in a 2,2-*cis* manner with two N atoms and two O atoms coordinating to the unique Rh^II^ atom *cis* to one another. The N_eq_—Rh—Rh—O_eq_ torsion angles on the acetamide bridge are 0.75 (7) and 1.99 (9)°. The axial nitrile ligand completes the distorted octa­hedral coordination sphere of one Rh^II^ atom and shows a nonlinear coordination, with an Rh—N—C bond angle of 162.8 (5)°; the N—C bond length is 1.154 (7) Å.

## Related literature   

For the synthesis and structure of four related compounds, see: Lifsey *et al.* (1987 ▶); Eagle *et al.* (2000 ▶, 2012 ▶, 2013*a* ▶,*b* ▶).
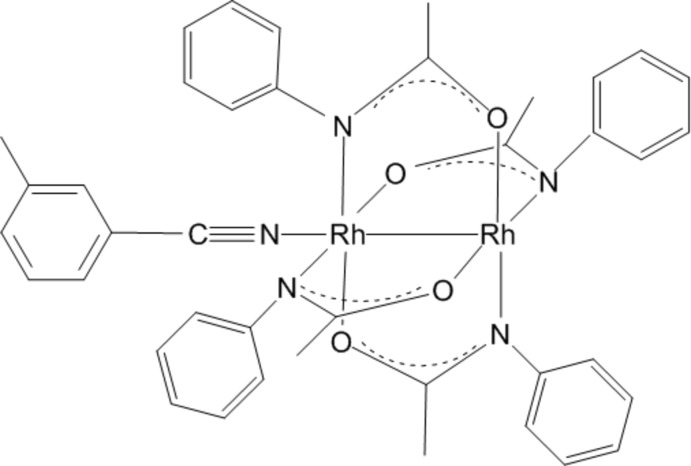


## Experimental   

### 

#### Crystal data  

[Rh_2_(C_8_H_8_NO)_4_(C_8_H_7_N)]
*M* *_r_* = 859.59
Orthorhombic, 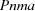

*a* = 15.3319 (14) Å
*b* = 18.3248 (16) Å
*c* = 12.9564 (12) Å
*V* = 3640.2 (6) Å^3^
*Z* = 4
Mo *K*α radiation
μ = 0.95 mm^−1^
*T* = 223 K
0.17 × 0.15 × 0.14 mm

#### Data collection  

Rigaku XtaLAB mini diffractometer
Absorption correction: multi-scan (*REQAB*; Rigaku, 1998 ▶) *T*_min_ = 0.664, *T*_max_ = 0.873
36328 measured reflections
4292 independent reflections
3154 reflections with *I* > 2σ(*I*)
*R*_int_ = 0.086

#### Refinement  

*R*[*F* ^2^ > 2σ(*F* ^2^)] = 0.042
*wR*(*F* ^2^) = 0.089
*S* = 1.05
4292 reflections
250 parameters
H-atom parameters constrained
Δρ_max_ = 0.75 e Å^−3^
Δρ_min_ = −0.50 e Å^−3^

### 

Data collection: *PROCESS-AUTO* (Rigaku, 2010 ▶); cell refinement: *PROCESS-AUTO*; data reduction: *PROCESS-AUTO*; program(s) used to solve structure: *SIR92* (Altomare *et al.*, 1994 ▶); program(s) used to refine structure: *SHELXL97* (Sheldrick, 2008 ▶); molecular graphics: *CrystalStructure* (Rigaku, 2010 ▶); software used to prepare material for publication: *CrystalStructure*.

## Supplementary Material

Crystal structure: contains datablock(s) General, I. DOI: 10.1107/S1600536814016031/mw2125sup1.cif

Structure factors: contains datablock(s) I. DOI: 10.1107/S1600536814016031/mw2125Isup2.hkl

CCDC reference: 1013071

Additional supporting information:  crystallographic information; 3D view; checkCIF report

## Figures and Tables

**Fig. 1.**
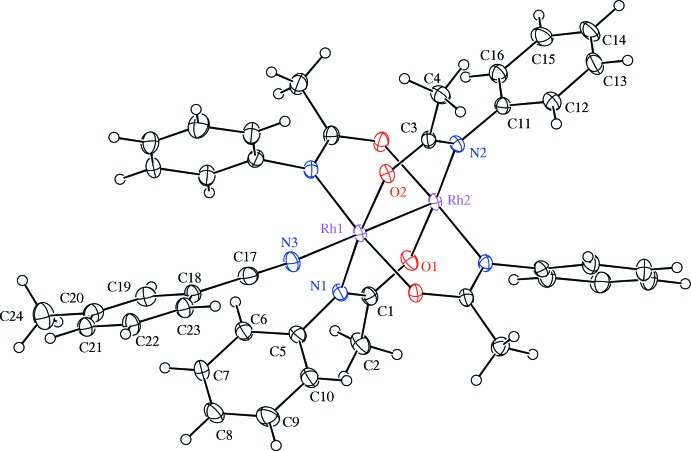
ORTEP of the title compound with 30% probability ellipsoids. Hydrogen atoms are drawn as small spheres.

## supplementary crystallographic information

### S2.1. Synthesis and crystallization

Approximately 10mg of *cis*-tetra­kis[µ-N-(phenyl)­acetamidato]-κ^4^N:O;κ^4^O:N dirhodium(II)] was dissolved in 18 mL of di­chloro­methane. 4µL of neat 3-methyl benzo­nitrile and 2µL of acetone were then added to this solution *via* a gas-tight syringe turning the solution from forest green to dark blue. Crystals grew over a two week period *via* vapor diffusion. From the structure determination compound 1 is an adduct of *cis*-tetra­kis[µ-N-(phenyl)­acetamidato]-κ^4^N:O;κ^4^O:N rhodium(II)] with 3-methyl benzo­nitrile in one axial site.

### S2.2. Refinement

H-atoms were included in calculated positions with C—H = 0.93 - 0.96 and included as riding contributions with isotropic displacement parameters 1.2-1.5 times those of the attached atom. H-atoms attached to C24 are disordered across the mirror plane.

The second parameter on the SHELXL weighting line has a large value (7.37) which may arise from inadequacies in the absorption correction.

### S3. Results and discussion

Previous papers report the structures of the related complexes 2,2-*trans*-Rh_2_[N(C_6_H_5_)COCH_3_]_4_·2NCC_6_H_5_ (2) (Eagle *et al.*, 2000), 2,2-*trans*-Rh_2_[N(C_9_H_11_)COCH_3_]_4_·2NCC_6_H_5_ (3) (Eagle *et al.*, 2012), 2,2-*cis*-[Rh_2_(N(C_6_H_5_)COCH_3_)_4_]·2NCC_6_H_5_ (4) (Eagle *et al.*, 2013*b*) and 2,2-*trans*-Rh_2_[N(C_6_H_5_)COCH_3_]_4_·NCC_7_H_7_ (5) (Eagle *et al.*, 2013*a*). The numbering scheme of the title compound is adopted from that of compound 2.

The axial rhodium-nitro­gen-carbon bond angle for 1, 162.8 (5)° (Fig.1) is distinctly non-linear which is different from those found in compound 2 (178.5 (5)° and 169.3 (5)°), and compound 3 (180°; imposed by space group symmetry), but similar to those found in compound 4 (167.14 (15)°) and compound 5 (166.4 (4)°). The axial carbon–nitro­gen bond length in 1 is 1.154 (7) Å which is comparable to corresponding distances found in 2 (1.135 (8) Å and 1.145 (8) Å) as well as 4 (1.135 (3) Å) and 5 (1.135 (3) Å) and slightly longer than 3 (1.106 (6) Å). The [Rh_2_[N(C_6_H_5_)COCH_3_)_4_] portion of compound 1 has approximate -4 symmetry with non-eclipsed N_eq_–Rh–Rh–O_eq_ torsion angles around each acetamide bridge of 0.75 (7)° or 1.99 (9)°. These can be compared to the range of 9.03° and 11.89° in 2 , 1.12 (9)° in 3, the range between 1.62 (4)° and 1.78 (4)° in 4 and 12.55 (11)° or 14.04 (8)° in 5. There are no unusually short inter­molecular distances.

The infrared absorption spectum of compound 1 showed bands at 2338 cm^-1^ and 2359 cm^-1^ attributable to carbon–nitro­gen bond stretching modes. The corresponding band for uncomplexed 3-methyl­benzo­nitrile appears at 2228 cm^-1^. This indicates that there is a shortening of the carbon–nitro­gen bond and a stronger σ-inter­action with the rhodium metal compared to the π-back bonding which occurs upon complexation with *trans*-tetra­kis[µ-N-(phenyl)­acetamidato]-κ^4^N:O;κ^4^O:N rhodium(II)].

### Crystal data

**Table:**

[Rh_2_(C_8_H_8_NO)_4_(C_8_H_7_N)]	*F*(000) = 1744.00
*M**_r_* = 859.59	*D*_x_ = 1.568 Mg m^−^^3^
Orthorhombic, *P**n**m**a*	Mo *K*α radiation, λ = 0.71075 Å
Hall symbol: -P 2ac 2n	Cell parameters from 27167 reflections
*a* = 15.3319 (14) Å	θ = 3.0–27.6°
*b* = 18.3248 (16) Å	µ = 0.95 mm^−^^1^
*c* = 12.9564 (12) Å	*T* = 223 K
*V* = 3640.2 (6) Å^3^	Chunk, green
*Z* = 4	0.17 × 0.15 × 0.14 mm

### Data collection

**Table:**

Rigaku XtaLAB mini diffractometer	3154 reflections with *I* > 2σ(*I*)
Detector resolution: 6.849 pixels mm^-1^	*R*_int_ = 0.086
ω scans	θ_max_ = 27.5°, θ_min_ = 3.0°
Absorption correction: multi-scan (*REQAB*; Rigaku, 1998)	*h* = −19→19
*T*_min_ = 0.664, *T*_max_ = 0.873	*k* = −23→23
36328 measured reflections	*l* = −16→16
4292 independent reflections	

### Refinement

**Table:**

Refinement on *F*^2^	Primary atom site location: structure-invariant direct methods
Least-squares matrix: full	Secondary atom site location: difference Fourier map
*R*[*F*^2^ > 2σ(*F*^2^)] = 0.042	Hydrogen site location: inferred from neighbouring sites
*wR*(*F*^2^) = 0.089	H-atom parameters constrained
*S* = 1.05	*w* = 1/[σ^2^(*F*_o_^2^) + (0.0277*P*)^2^ + 7.3733*P*] where *P* = (*F*_o_^2^ + 2*F*_c_^2^)/3
4292 reflections	(Δ/σ)_max_ = 0.005
250 parameters	Δρ_max_ = 0.75 e Å^−^^3^
0 restraints	Δρ_min_ = −0.50 e Å^−^^3^

### Special details

**Table:**

Geometry. Compound 1 is coordinated by 3-methyl benzonitrile to only one axial site. In compounds 2 through 4 there are no methyl groups on the benzonitrile ligand and each of them has a benzonitrile ligand attached in each axial site. Like compound 1, compound 5 is coordinated by 3-methyl benzonitrile to only one axial site, however compound 5 exists as the *trans*-acetamide isomer, whereas compound 1 is the *cis*-acetamide isomer. The predominance of σ-bonding in the rhodium-nitrogen-carbon bond system (and lower affect of π-back bonding) is the likely cause of this deviation from linearity for compound 1, which has a similar rhodium-nitrogen-carbon angle as compound 5. The packing diagram shows that two acetamide phenyl rings on the same rhodium are stacked upon each other.
Refinement. Refinement was performed using all reflections. The weighted *R*-factor (*wR*) and goodness of fit (*S*) are based on *F*^2^. *R*-factor (gt) are based on *F*. The threshold expression of *F*^2^ > 2.0 σ(*F*^2^) is used only for calculating *R*-factor (gt).

### Fractional atomic coordinates and isotropic or equivalent isotropic displacement parameters (Å^2^)

**Table:**

	*x*	*y*	*z*	*U*_iso_*/*U*_eq_	Occ. (<1)
Rh1	0.47995 (2)	0.2500	0.52236 (3)	0.02157 (11)	
Rh2	0.32933 (2)	0.2500	0.46954 (3)	0.02139 (11)	
O1	0.35639 (16)	0.32962 (14)	0.36333 (19)	0.0294 (6)	
O2	0.44972 (15)	0.16995 (14)	0.62786 (18)	0.0254 (6)	
N1	0.49816 (19)	0.33240 (17)	0.4172 (2)	0.0262 (7)	
N2	0.30842 (19)	0.17003 (16)	0.5753 (2)	0.0237 (6)	
N3	0.6160 (3)	0.2500	0.5704 (4)	0.0336 (11)	
C1	0.4333 (2)	0.3578 (2)	0.3624 (3)	0.0273 (8)	
C2	0.4427 (3)	0.4243 (2)	0.2942 (3)	0.0401 (10)	
H2A	0.4958	0.4501	0.3121	0.048*	
H2B	0.3930	0.4562	0.3043	0.048*	
H2C	0.4453	0.4092	0.2226	0.048*	
C3	0.3715 (2)	0.1440 (2)	0.6319 (3)	0.0252 (8)	
C4	0.3608 (3)	0.0811 (2)	0.7052 (3)	0.0342 (9)	
H4A	0.4090	0.0473	0.6966	0.041*	
H4B	0.3064	0.0562	0.6908	0.041*	
H4C	0.3602	0.0991	0.7756	0.041*	
C5	0.5811 (2)	0.3681 (2)	0.4141 (3)	0.0283 (8)	
C6	0.6393 (3)	0.3544 (2)	0.3362 (3)	0.0342 (9)	
H6	0.6232	0.3231	0.2819	0.041*	
C7	0.7218 (3)	0.3860 (2)	0.3363 (3)	0.0386 (10)	
H7	0.7611	0.3766	0.2822	0.046*	
C8	0.7452 (3)	0.4311 (2)	0.4159 (4)	0.0428 (11)	
H8	0.8008	0.4527	0.4163	0.051*	
C9	0.6879 (3)	0.4451 (3)	0.4957 (4)	0.0457 (11)	
H9	0.7047	0.4755	0.5506	0.055*	
C10	0.6056 (3)	0.4140 (2)	0.4942 (3)	0.0373 (10)	
H10	0.5661	0.4240	0.5478	0.045*	
C11	0.2218 (2)	0.1406 (2)	0.5787 (3)	0.0270 (8)	
C12	0.1718 (3)	0.1409 (2)	0.6679 (3)	0.0344 (9)	
H12	0.1944	0.1608	0.7291	0.041*	
C13	0.0885 (3)	0.1117 (2)	0.6669 (4)	0.0423 (11)	
H13	0.0555	0.1112	0.7280	0.051*	
C14	0.0535 (3)	0.0835 (2)	0.5780 (4)	0.0464 (12)	
H14	−0.0028	0.0633	0.5784	0.056*	
C15	0.1015 (3)	0.0850 (2)	0.4881 (4)	0.0424 (11)	
H15	0.0773	0.0668	0.4266	0.051*	
C16	0.1855 (3)	0.1134 (2)	0.4879 (3)	0.0334 (9)	
H16	0.2180	0.1142	0.4263	0.040*	
C17	0.6913 (4)	0.2500	0.5696 (5)	0.0339 (13)	
C18	0.7854 (3)	0.2500	0.5655 (5)	0.0295 (12)	
C19	0.8285 (4)	0.2500	0.4693 (4)	0.0331 (13)	
H19	0.7953	0.2500	0.4083	0.040*	
C20	0.9168 (4)	0.2500	0.4626 (4)	0.0356 (14)	
C21	0.9642 (4)	0.2500	0.5533 (4)	0.0315 (13)	
H21	1.0255	0.2500	0.5501	0.038*	
C22	0.9239 (4)	0.2500	0.6481 (4)	0.0354 (14)	
H22	0.9578	0.2500	0.7085	0.042*	
C23	0.8346 (4)	0.2500	0.6552 (4)	0.0317 (13)	
H23	0.8072	0.2500	0.7200	0.038*	
C24	0.9623 (5)	0.2500	0.3590 (5)	0.0500 (18)	
H24A	0.9234	0.2697	0.3070	0.060*	0.5
H24B	0.9783	0.2004	0.3407	0.060*	0.5
H24C	1.0144	0.2799	0.3630	0.060*	0.5

### Atomic displacement parameters (Å^2^)

**Table:**

	*U*^11^	*U*^22^	*U*^33^	*U*^12^	*U*^13^	*U*^23^
Rh1	0.0146 (2)	0.0287 (2)	0.0214 (2)	0.000	0.00038 (16)	0.000
Rh2	0.0151 (2)	0.0283 (2)	0.0207 (2)	0.000	−0.00015 (16)	0.000
O1	0.0226 (14)	0.0381 (15)	0.0275 (13)	−0.0035 (12)	−0.0011 (11)	0.0101 (12)
O2	0.0185 (13)	0.0331 (15)	0.0245 (13)	−0.0011 (11)	0.0004 (10)	0.0033 (11)
N1	0.0205 (16)	0.0297 (17)	0.0284 (16)	−0.0020 (13)	0.0027 (13)	0.0021 (14)
N2	0.0173 (15)	0.0270 (16)	0.0267 (15)	−0.0019 (13)	−0.0004 (13)	0.0021 (14)
N3	0.018 (2)	0.040 (3)	0.043 (3)	0.000	−0.004 (2)	0.000
C1	0.025 (2)	0.034 (2)	0.0230 (18)	−0.0017 (17)	0.0019 (16)	0.0006 (16)
C2	0.031 (2)	0.044 (3)	0.045 (2)	−0.004 (2)	0.001 (2)	0.014 (2)
C3	0.0210 (19)	0.029 (2)	0.0253 (18)	−0.0006 (16)	0.0037 (15)	−0.0027 (16)
C4	0.029 (2)	0.033 (2)	0.041 (2)	−0.0041 (18)	−0.0036 (18)	0.0046 (19)
C5	0.025 (2)	0.029 (2)	0.031 (2)	−0.0025 (16)	0.0002 (16)	0.0043 (17)
C6	0.031 (2)	0.037 (2)	0.034 (2)	−0.0065 (19)	0.0060 (18)	−0.0054 (19)
C7	0.024 (2)	0.040 (2)	0.052 (3)	−0.0002 (18)	0.0121 (19)	0.000 (2)
C8	0.027 (2)	0.044 (3)	0.058 (3)	−0.008 (2)	−0.002 (2)	0.003 (2)
C9	0.044 (3)	0.048 (3)	0.046 (3)	−0.014 (2)	−0.004 (2)	−0.009 (2)
C10	0.033 (2)	0.042 (2)	0.037 (2)	−0.0073 (19)	0.0066 (18)	−0.004 (2)
C11	0.0205 (19)	0.026 (2)	0.034 (2)	0.0012 (15)	−0.0014 (16)	0.0012 (18)
C12	0.027 (2)	0.037 (2)	0.039 (2)	−0.0050 (18)	0.0002 (18)	−0.0017 (19)
C13	0.028 (2)	0.045 (3)	0.053 (3)	−0.003 (2)	0.010 (2)	0.008 (2)
C14	0.021 (2)	0.041 (3)	0.077 (3)	−0.0094 (19)	−0.001 (2)	0.007 (3)
C15	0.030 (2)	0.038 (2)	0.059 (3)	−0.0072 (19)	−0.011 (2)	−0.002 (2)
C16	0.029 (2)	0.037 (2)	0.034 (2)	−0.0027 (17)	−0.0006 (17)	−0.0021 (19)
C17	0.032 (3)	0.030 (3)	0.040 (3)	0.000	−0.006 (3)	0.000
C18	0.019 (3)	0.030 (3)	0.040 (3)	0.000	−0.004 (2)	0.000
C19	0.034 (3)	0.035 (3)	0.030 (3)	0.000	−0.007 (3)	0.000
C20	0.039 (3)	0.032 (3)	0.035 (3)	0.000	−0.001 (3)	0.000
C21	0.027 (3)	0.035 (3)	0.033 (3)	0.000	0.000 (2)	0.000
C22	0.024 (3)	0.048 (4)	0.034 (3)	0.000	−0.005 (2)	0.000
C23	0.032 (3)	0.040 (3)	0.024 (3)	0.000	0.002 (2)	0.000
C24	0.050 (4)	0.067 (5)	0.033 (3)	0.000	0.008 (3)	0.000

### Geometric parameters (Å, º)

**Table:**

Rh1—N1	2.053 (3)	C8—C9	1.380 (6)
Rh1—N1^i^	2.053 (3)	C8—H8	0.9400
Rh1—O2^i^	2.058 (2)	C9—C10	1.385 (6)
Rh1—O2	2.058 (2)	C9—H9	0.9400
Rh1—N3	2.177 (4)	C10—H10	0.9400
Rh1—Rh2	2.4086 (6)	C11—C12	1.387 (5)
Rh2—N2^i^	2.032 (3)	C11—C16	1.394 (5)
Rh2—N2	2.032 (3)	C12—C13	1.384 (5)
Rh2—O1	2.048 (2)	C12—H12	0.9400
Rh2—O1^i^	2.048 (2)	C13—C14	1.371 (6)
O1—C1	1.288 (4)	C13—H13	0.9400
O2—C3	1.290 (4)	C14—C15	1.378 (6)
N1—C1	1.308 (5)	C14—H14	0.9400
N1—C5	1.431 (5)	C15—C16	1.389 (5)
N2—C3	1.304 (5)	C15—H15	0.9400
N2—C11	1.434 (4)	C16—H16	0.9400
N3—C17	1.154 (7)	C17—C18	1.444 (8)
C1—C2	1.511 (5)	C18—C23	1.385 (7)
C2—H2A	0.9700	C18—C19	1.411 (8)
C2—H2B	0.9700	C19—C20	1.356 (8)
C2—H2C	0.9700	C19—H19	0.9400
C3—C4	1.503 (5)	C20—C21	1.381 (8)
C4—H4A	0.9700	C20—C24	1.513 (8)
C4—H4B	0.9700	C21—C22	1.375 (8)
C4—H4C	0.9700	C21—H21	0.9400
C5—C6	1.370 (5)	C22—C23	1.372 (8)
C5—C10	1.387 (5)	C22—H22	0.9400
C6—C7	1.392 (5)	C23—H23	0.9400
C6—H6	0.9400	C24—H24A	0.9700
C7—C8	1.370 (6)	C24—H24B	0.9700
C7—H7	0.9400	C24—H24C	0.9700
			
N1—Rh1—N1^i^	94.72 (17)	C7—C6—H6	119.5
N1—Rh1—O2^i^	86.96 (11)	C8—C7—C6	119.3 (4)
N1^i^—Rh1—O2^i^	174.72 (11)	C8—C7—H7	120.3
N1—Rh1—O2	174.72 (11)	C6—C7—H7	120.3
N1^i^—Rh1—O2	86.96 (11)	C7—C8—C9	120.7 (4)
O2^i^—Rh1—O2	90.92 (14)	C7—C8—H8	119.7
N1—Rh1—N3	93.41 (12)	C9—C8—H8	119.7
N1^i^—Rh1—N3	93.41 (12)	C8—C9—C10	119.5 (4)
O2^i^—Rh1—N3	91.48 (11)	C8—C9—H9	120.2
O2—Rh1—N3	91.48 (11)	C10—C9—H9	120.2
N1—Rh1—Rh2	86.66 (8)	C9—C10—C5	120.4 (4)
N1^i^—Rh1—Rh2	86.66 (8)	C9—C10—H10	119.8
O2^i^—Rh1—Rh2	88.44 (7)	C5—C10—H10	119.8
O2—Rh1—Rh2	88.44 (7)	C12—C11—C16	118.9 (3)
N3—Rh1—Rh2	179.89 (13)	C12—C11—N2	122.5 (3)
N2^i^—Rh2—N2	92.30 (17)	C16—C11—N2	118.6 (3)
N2^i^—Rh2—O1	88.36 (11)	C13—C12—C11	120.1 (4)
N2—Rh2—O1	177.38 (11)	C13—C12—H12	120.0
N2^i^—Rh2—O1^i^	177.38 (11)	C11—C12—H12	120.0
N2—Rh2—O1^i^	88.36 (11)	C14—C13—C12	121.0 (4)
O1—Rh2—O1^i^	90.86 (15)	C14—C13—H13	119.5
N2^i^—Rh2—Rh1	87.68 (8)	C12—C13—H13	119.5
N2—Rh2—Rh1	87.68 (8)	C13—C14—C15	119.6 (4)
O1—Rh2—Rh1	89.81 (7)	C13—C14—H14	120.2
O1^i^—Rh2—Rh1	89.81 (7)	C15—C14—H14	120.2
C1—O1—Rh2	118.6 (2)	C14—C15—C16	120.3 (4)
C3—O2—Rh1	119.9 (2)	C14—C15—H15	119.9
C1—N1—C5	119.8 (3)	C16—C15—H15	119.9
C1—N1—Rh1	121.3 (2)	C15—C16—C11	120.1 (4)
C5—N1—Rh1	118.4 (2)	C15—C16—H16	119.9
C3—N2—C11	122.2 (3)	C11—C16—H16	119.9
C3—N2—Rh2	121.7 (2)	N3—C17—C18	178.5 (7)
C11—N2—Rh2	116.0 (2)	C23—C18—C19	119.0 (5)
C17—N3—Rh1	162.8 (5)	C23—C18—C17	120.9 (5)
O1—C1—N1	123.2 (3)	C19—C18—C17	120.0 (5)
O1—C1—C2	114.6 (3)	C20—C19—C18	121.6 (5)
N1—C1—C2	122.2 (3)	C20—C19—H19	119.2
C1—C2—H2A	109.5	C18—C19—H19	119.2
C1—C2—H2B	109.5	C19—C20—C21	118.1 (6)
H2A—C2—H2B	109.5	C19—C20—C24	121.1 (6)
C1—C2—H2C	109.5	C21—C20—C24	120.8 (5)
H2A—C2—H2C	109.5	C22—C21—C20	121.5 (5)
H2B—C2—H2C	109.5	C22—C21—H21	119.2
O2—C3—N2	122.1 (3)	C20—C21—H21	119.2
O2—C3—C4	114.2 (3)	C23—C22—C21	120.6 (5)
N2—C3—C4	123.7 (3)	C23—C22—H22	119.7
C3—C4—H4A	109.5	C21—C22—H22	119.7
C3—C4—H4B	109.5	C22—C23—C18	119.2 (5)
H4A—C4—H4B	109.5	C22—C23—H23	120.4
C3—C4—H4C	109.5	C18—C23—H23	120.4
H4A—C4—H4C	109.5	C20—C24—H24A	109.5
H4B—C4—H4C	109.5	C20—C24—H24B	109.5
C6—C5—C10	119.1 (4)	H24A—C24—H24B	109.5
C6—C5—N1	121.0 (4)	C20—C24—H24C	109.5
C10—C5—N1	119.8 (3)	H24A—C24—H24C	109.5
C5—C6—C7	121.0 (4)	H24B—C24—H24C	109.5
C5—C6—H6	119.5		
			
Rh2—O1—C1—N1	8.4 (5)	N1—C5—C10—C9	175.7 (4)
Rh2—O1—C1—C2	−170.2 (2)	C3—N2—C11—C12	61.3 (5)
C5—N1—C1—O1	−178.2 (3)	Rh2—N2—C11—C12	−122.9 (3)
Rh1—N1—C1—O1	−6.1 (5)	C3—N2—C11—C16	−121.2 (4)
C5—N1—C1—C2	0.3 (5)	Rh2—N2—C11—C16	54.6 (4)
Rh1—N1—C1—C2	172.4 (3)	C16—C11—C12—C13	2.6 (6)
Rh1—O2—C3—N2	−4.4 (5)	N2—C11—C12—C13	−179.9 (4)
Rh1—O2—C3—C4	174.4 (2)	C11—C12—C13—C14	−1.3 (7)
C11—N2—C3—O2	179.1 (3)	C12—C13—C14—C15	−0.8 (7)
Rh2—N2—C3—O2	3.6 (5)	C13—C14—C15—C16	1.4 (7)
C11—N2—C3—C4	0.4 (5)	C14—C15—C16—C11	−0.1 (6)
Rh2—N2—C3—C4	−175.1 (3)	C12—C11—C16—C15	−1.9 (6)
C1—N1—C5—C6	−82.5 (5)	N2—C11—C16—C15	−179.5 (4)
Rh1—N1—C5—C6	105.1 (4)	C23—C18—C19—C20	0.000 (1)
C1—N1—C5—C10	101.5 (4)	C17—C18—C19—C20	180.000 (1)
Rh1—N1—C5—C10	−70.9 (4)	C18—C19—C20—C21	0.000 (1)
C10—C5—C6—C7	−0.4 (6)	C18—C19—C20—C24	180.000 (1)
N1—C5—C6—C7	−176.4 (4)	C19—C20—C21—C22	0.000 (1)
C5—C6—C7—C8	0.5 (7)	C24—C20—C21—C22	180.000 (1)
C6—C7—C8—C9	0.1 (7)	C20—C21—C22—C23	0.000 (1)
C7—C8—C9—C10	−0.9 (7)	C21—C22—C23—C18	0.000 (1)
C8—C9—C10—C5	1.1 (7)	C19—C18—C23—C22	0.000 (1)
C6—C5—C10—C9	−0.4 (6)	C17—C18—C23—C22	180.000 (1)

Symmetry code: (i) *x*, −*y*+1/2, *z*.

## References

[bb1] Altomare, A., Cascarano, G., Giacovazzo, C., Guagliardi, A., Burla, M. C., Polidori, G. & Camalli, M. (1994). *J. Appl. Cryst.* **27**, 435.

[bb2] Eagle, C. T., Atem-Tambe, N., Kpogo, K. K., Tan, J. & Quarshie, F. (2013*a*). *Acta Cryst.* E**69**, m639. 10.1107/S1600536813029838 PMC3884982 24454157

[bb3] Eagle, C. T., Farrar, D. G., Holder, G. N., Pennington, W. T. & Bailey, R. D. (2000). *J. Organomet. Chem* **596**, 90–94.

[bb4] Eagle, C. T., Kpogo, K. K., Zink, L. C. & Smith, A. E. (2012). *Acta Cryst.* E**68**, m877. 10.1107/S1600536812024518 PMC3393156 22807724

[bb5] Eagle, C. T., Quarshie, F., Ketron, M. E. & Atem-Tambe, N. (2013*b*). *Acta Cryst.* E**69**, m329. 10.1107/S1600536813012828 PMC3684889 23794991

[bb6] Lifsey, R. S., Lin, X. Q., Chavan, M. Y., Ahsan, M. Q., Kadish, K. M. & Bear, J. L. (1987). *Inorg. Chem.* **26**, 830–836.

[bb7] Rigaku (1998). *REQAB* Rigaku Americas, The Woodlands, Texas, USA, and Rigaku Corporation, Tokyo, Japan.

[bb8] Rigaku (2010). *CrystalStructure* and *PROCESS-AUTO* Rigaku Corporation, Tokyo, Japan.

[bb9] Sheldrick, G. M. (2008). *Acta Cryst.* A**64**, 112–122. 10.1107/S0108767307043930 18156677

